# Examination of Spatial Polygamy among Young Gay, Bisexual, and Other Men Who Have Sex with Men in New York City: The P18 Cohort Study

**DOI:** 10.3390/ijerph110908962

**Published:** 2014-08-28

**Authors:** Dustin T. Duncan, Farzana Kapadia, Perry N. Halkitis

**Affiliations:** 1 Department of Population Health, School of Medicine, New York University, New York, NY 10016, USA; E-Mails: farzana.kapadia@nyu.edu (F.K.); perry.halkitis@nyu.edu (P.N.K.); 2 Global Institute of Public Health, New York University, New York, NY 10003, USA; 3 Population Center, New York University, New York, NY 10012, USA; 4 Center for Health, Identity, Behavior and Prevention Studies, New York University, New York, NY 10003, USA; 5 Department of Nutrition, Food Studies and Public Health, Steinhardt School of Culture, Education and Human Development, New York University, New York, NY 10003, USA; 6 Department of Applied Psychology, Steinhardt School of Culture, Education and Human Development, New York University, New York, NY 10003, USA

**Keywords:** spatial polygamy, neighborhoods, gay men’s health

## Abstract

The few previous studies examining the influence of the neighborhood context on health and health behavior among young gay, bisexual, and other men who have sex with men (YMSM) have predominantly focused on residential neighborhoods. No studies have examined multiple neighborhood contexts among YMSM or the relationships between sociodemographic characteristics, psychosocial factors, social support network characteristics, health behaviors, and neighborhood concordance. In this study, we assessed spatial polygamy by determining the amount of concordance between residential, social, and sex neighborhoods (defined as boroughs) in addition to examining individual-level characteristics that may be associated with neighborhood concordance. These data come from the baseline assessment of Project 18, a cohort of racially and ethnically diverse YMSM residing in the New York City metropolitan area. Participants (N = 598) provided information on their residential, social, and sex boroughs as well as information on their sociodemographic characteristics, psychosocial factors, social support network characteristics, and health behaviors (e.g., substance use and condomless sex). Descriptive analyses were conducted to examine the distribution of boroughs reported across all three contexts,* i.e.*, residential, social, and sex boroughs. Next, concordance between: (1) residential and social boroughs; (2) residential and sex boroughs; (3) social and sex boroughs; and (4) residential, social, and sex boroughs was assessed. Finally, bivariable analyses were conducted to examine the relationships between sociodemographic characteristics, psychosocial factors, social support network characteristics, and health behaviors in relation to borough concordance. Approximately two-thirds of participants reported concordance between residential/socializing, residential/sex, and sex/socializing boroughs, whereas 25% reported concordance between all three residential/socializing/sex boroughs. Borough concordance varied by some individual-level characteristics. For example, White YMSM and YMSM reporting lower perceived socioeconomic status were significantly more likely to report residential/socializing/sex borough concordance (*p* < 0.001). With regard to psychosocial factors, YMSM who reported experiencing gay-related stigma in public forums were more likely to report discordant socializing/sex and residential/socializing/sex boroughs (*p* < 0.001). Greater frequency of communication with network members (≥weekly) was associated with less residential/social borough concordance (*p* < 0.05). YMSM who reported residential/socializing/sex borough concordance were more likely to report recent (last 30 days) alcohol use, recent marijuana use, and recently engaging in condomless oral sex (all *p* < 0.05). These findings suggest that spatial polygamy, or an individual moving across and experiencing multiple neighborhood contexts, is prevalent among urban YMSM and that spatial polygamy varies by multiple individual-level characteristics. Future research among YMSM populations should consider multiple neighborhood contexts in order to provide a more nuanced understanding of how and which neighborhood contexts influence the health and well-being of YMSM. This further examination of spatial polygamy (and individual-level characteristics associated with it) may increase understanding of the most appropriate locations for targeted disease prevention and health promotion interventions (e.g., HIV prevention interventions).

## 1. Introduction

Disparities in health and health behaviors—including substance use, condomless sexual behaviors and HIV outcomes—persist among gay, bisexual, and other men who have sex with men (MSM) despite decades of behavioral research examining a broad range of individual-level factors. Moving beyond studies of individual-level factors, a focus on social and spatial contexts may help explain variation in health and health behaviors in MSM populations. Emerging research shows that neighborhoods can play an important role in influencing health and health behaviors (e.g., substance use and condomless sex) among MSM [1,2,3,4,5]. Similar to research in general (*i.e.*, non-sexual minority) populations, existing research on neighborhoods and health among MSM populations suffer from a predominant focus on only one type of neighborhood environment or spatial context (*i.e.*, the residential neighborhood). However, people move through multiple spatial contexts (e.g., travel between home, school, church, work, shopping centers, and socializing locations) [6]. As such, the exclusive focus on the residential environment (*i.e.*, the “residential trap”) is problematic because it fails to capture the diversity of spatial contexts that one may experience [7]. For MSM, in particular, other spatial contexts—such as neighborhoods where MSM socialize and neighborhoods where MSM engage in sexual activity—may be salient and important to their health and health behaviors. To illustrate, in one recent study among young gay, bisexual, and other MSM in New York City, the majority of the participants socialized outside of their home neighborhoods and younger MSM often utilized their social circles to meet casual sex partners [8]. 

As originally conceptualized by Dr. John Odland (from Indiana University) and recently discussed by Dr. Stephen Matthews (from Penn State University), the concept of spatial polygamy not only asserts that individuals experience and interact in various contexts, but also that these varied spatial contexts influence and may be differentially associated with shaping health outcomes [6]. Thus, spatial polygamy may be more appropriate for considering whether and how different spatial contexts influence the health of MSM. For example, spatial polygamy may be linked to MSM health because those who travel to multiple neighborhoods could engage in sexual activity in these different contexts, increasing their HIV risk (especially if they engage in high-risk sexual behaviors across neighborhoods with a high HIV prevalence). While little is known about spatial polygamy and salient spatial contexts in general populations, and especially so for MSM populations, the extant research suggests that MSM move across spatial contexts [9,10,11]. For example, a qualitative study showed that MSM in New York City move through different neighborhoods—including to maintain connections with their various networks (e.g., their family and sexual networks) [11]. Interestingly and importantly, a recent study by Koblin and colleagues [10] of 706 MSM with an average age of approximately 32 years old who were from the New York City boroughs found that only 15% had concordant home, social, and sexual neighborhoods and that 31% of these men reported that none of their neighborhoods were the same [10]. In that study, concordance of neighborhoods differed significantly by race/ethnicity; Black and Latino men had less neighborhood concordance for all of their neighborhood types compared with their White counterparts [10]. 

While previous research on spatial polygamy among MSM populations is informative, there is a need for additional research to examine spatial polygamy in order to assess the extent that spatial polygamy may exist in other MSM populations (including young men who have sex with men (YMSM)), which may be different from MSM populations already studied. Compared to adult MSM, YMSM might be more spatially mobile because they may be more curious and more interested in exploration, including exploring different neighborhoods. In addition, YMSM might be more likely than adult MSM to leave their residential neighborhoods because they want to socialize in gay friendly neighborhoods (where they can be more open about their sexuality), increasing their spatial polygamy. Moreover, YMSM might be more spatially mobile, due to being more physically active, than older and elderly MSM populations, especially older and elderly MSM who may have a disability [12,13], and thus YMSM may be more likely to engage in more spatial polygamy. Furthermore, additional research is needed to examine whether sociodemographic characteristics, psychosocial factors, social support network characteristics, and health behaviors are associated with neighborhood concordance, which (to our best knowledge) has not been done to date. However, conceptual models linking neighborhood environments to health risk behaviors and related outcomes (including alcohol and drug use and sexual risk behavior) among MSM underscore that salient features could be sociodemographic characteristics, psychosocial factors, and social support network characteristics, among others [14]. 

Examination of spatial polygamy (and individual-level characteristics associated with it) may help researchers, practitioners, and policymakers determine the most appropriate location(s) for targeted disease prevention and health promotion interventions (e.g., HIV prevention interventions). For example, examining psychosocial factors such as experiences with gay-related stigma that might be related to spatial polygamy (because living in a homophobic neighborhood might encourage someone to socialize in a non-homophobic/another neighborhood) could help us determine what types of individuals are more (or less) likely to engage in spatial polygamy. Thus, among a sample of a new generation of YMSM in the New York City metropolitan area including Westchester County, New Jersey and Connecticut, the present study sought to assess spatial polygamy by determining the amount of concordance between residential, social, and sex neighborhoods. In this study, we also evaluated whether sociodemographic characteristics, psychosocial factors, social support network characteristics, and health behaviors (*i.e.*, substance use and condomless sex) were associated with neighborhood concordance. New York City is an ideal location for this research because it is 304.8 square miles, with a population density of 27,440 persons per square mile, making it the most densely populated city in the United States. It contains 181 ZIP codes. Like other geographic locations, there are various ways to define a “neighborhood” in New York City and in this study we define neighborhoods as boroughs, due to the structure of the data. In New York City, there are five boroughs, each which is a county of New York State: The Bronx, Brooklyn, Manhattan, Queens, and Staten Island—which consolidated into a single city in 1898. 

## 2. Methods

### 2.1. Study Design and Overview

Data for this study come from the baseline assessment of Project 18, a longitudinal study of 598 racially and ethnically diverse YMSM in the New York City metropolitan area with complete data at baseline. Full details on the Project 18 study have been described elsewhere [15,16,17,18,19,20,21]. Briefly, participants were recruited via active and passive strategies between June 2009 and May 2011—including community events and dance clubs, as well as social networking websites and dating websites. Participants were eligible for this study if they were 18 or 19 years old at the time of the baseline assessment, biologically male, lived in the New York City metropolitan area, reported having had sex (any physical contact that could lead to orgasm) with another male in the 6 months preceding the baseline assessment, and self-reported a HIV-negative or unknown serostatus. Men from a racial/ethnic minority group (*i.e.*, Black, Hispanic, Asian-Pacific Islander, Multiracial/Other) were oversampled and thus were the majority of the sample (>70% non-White). The New York University Institutional Review Board approved the study protocol. 

#### 2.1.1. Characterization of Neighborhood Environments

In order to explore a range of neighborhood contexts that YMSM engage and interact in, this study sought to ascertain information about three different neighborhood contexts: the residential, social, and sex neighborhoods among study participants, which was based on our previous work highlighting these contexts as salient to MSM [8]. First, to characterize residential neighborhoods, participants were asked to report their current *ZIP code of residence*, which we geocoded in ArcGIS (Environmental Systems Research Institute (ESRI), Redlands, California, USA) based on procedures detailed in our prior work [22,23] using a ZIP code file from ESRI Data and Map current as of June 2011. Of the 598 participants who provided ZIP code data, 594 were geocoded while four were unable to be geocoded (due to invalid ZIP codes). In addition, all participants were asked to provide the names of their most recent (last 6 months) residential neighborhoods. Second, participants were asked to report on the neighborhoods where they had socialized most often during the last 6 months. Finally, participants were asked to provide names of the neighborhoods where they had sex most often during the past 6 months. All neighborhood information was obtained via survey. For each residential, social, and sex neighborhood, participants were asked to enter in the nearest cross streets or name of the neighborhood as opposed to geopolitical boundaries (e.g., ZIP codes). We did not obtain geopolitical boundaries for socializing and sex neighborhoods because we suspected this information might not be known for these neighborhoods in the same way that they are likely to be known for residential neighborhoods. Participants were asked to report the names of up to three neighborhoods for each neighborhood type (*i.e.*, residential, social, and sex neighborhoods); however, there was often no data for the second and third neighborhood names for each neighborhood type. It was not clear if the data were missing or not entered as participants may have only reported one or two neighborhoods. As such, we restricted the neighborhood information to the first neighborhood name for each type. In addition, because these data were self-reported, there was significant variation in how this information was entered (e.g., from specific cross street intersections in Manhattan to New York City boroughs, to cities outside of New York State). Given the variability in responses obtained for residential, social, and sex neighborhoods, the data on neighborhoods were coded as follows: New York City (metropolitan area, out of metropolitan area), New Jersey, Connecticut and other areas. The New York City metropolitan area included each New York City borough and its respective county: The Bronx (Bronx County), Brooklyn (Kings County), Manhattan (New York County), Queens (Queens County), and Staten Island (Richmond County). Any residential, social, or sex neighborhoods outside of the New York City metropolitan area, but within New York State, were categorized as outside of the New York City metropolitan area (e.g., Westchester County). Participants reporting social or sex neighborhoods in locations/cities outside of New York State, but within the tri-state area (*i.e.*, New Jersey and Connecticut), were categorized by that respective state. Finally, participants who reported social, or sex neighborhoods locations outside of the tri-state area (e.g., Los Angeles, California) were included in the “Other” category. Therefore, given the structure of the data, *characterization of neighborhoods for the spatial polygamy analyses was based on New York City boroughs*.

#### 2.1.2. Individual-Level Characteristics 

Individual-level characteristics were collected via audio computer-assisted self-interview (ACASI) software. A full description of these characteristics, including specific instruments used and their psychometric properties, has been described previously [15,16,17,18,19,20,21]. 

*Sociodemographic Characteristics.* The survey assessed race/ethnicity (Hispanic/Latino, Black, Asian-Pacific Islander/Multiracial/Other, and White), current school enrollment (yes, no), perceived familial socioeconomic status (lower, middle, upper), foreign-born status (yes, no), household composition (living with parents, friends/roommates, other (e.g., shelter)), sexual identity (exclusively homosexual* versus* not exclusively homosexual), currently in a relationship with another man (yes, no), and current housing status (stably housed* versus* unstably housed/homeless). 

*Psychosocial Factors*. Ethnic identity was measured using the 12-item Multigroup Ethnic Identity Measure (e.g., “I have a lot of pride in my ethnic group”,* etc.*) [24] (coded as low* versus* high), experiences of gay-related stigma in both public (coded as low* versus* high) and personal (coded as low* versus* high) contexts was assessed via two subscales of the HIV Stigma Scale that we modified to assess stigma associated with sexual orientation including a two-item measure (e.g., “Most people think a person who is gay is disgusting”) and via a three-item measure (e.g., “I have been hurt by how people reacted to learning I’m gay”), respectively [25], having come out to their peers as measured by the proportion of friends who knew of the participant’s same-sex sexual behavior (“About how many of your friends know that you have had sex with a man?” [26] (coded as no/“some, few, none, don’t know”* versus* yes/“all, most”), gay community affinity was assessed from a one-item measure: “I feel a part of the gay community in New York City” [26] (coded as high* versus* medium/low), and internalized homophobia from a four-item measure (e.g., “Sometimes I dislike myself for being gay/bisexual”) [27] (coded as low* versus* high) was assessed.

*Social Support Network Characteristics*. Social support network characteristics were assessed via a social network inventory of up to 10 network members (e.g., family, friends, work associates, sexual partner) who study participants considered to be a “significant or important” person in their lives, consistent with our previous work [16,21]. Network size (dichotomized at median as ≤8* versus* >8), average duration of network relationships (dichotomized as <2 years* versus* ≥2 years), frequency of communication with network members (dichotomized at ≤monthly* versus* ≥weekly), emotional support (low* versus* high), and material support (low* versus* high) were assessed. 

*Substance Use and Condomless Sex*. Participants provided information on recent substance use and condomless sex using a 30-day calendar-based methodology [28]. Specifically, recent (last 30 days) alcohol use and recent marijuana use were assessed in the survey. In addition, recent (last 30 days) condomless oral sex and condomless insertive or receptive anal sex were also assessed in the survey. Recent substance use and condomless sex was coded was “yes”* versus* “no”. 

### 2.2. Statistical Analyses

**Figure 1:**
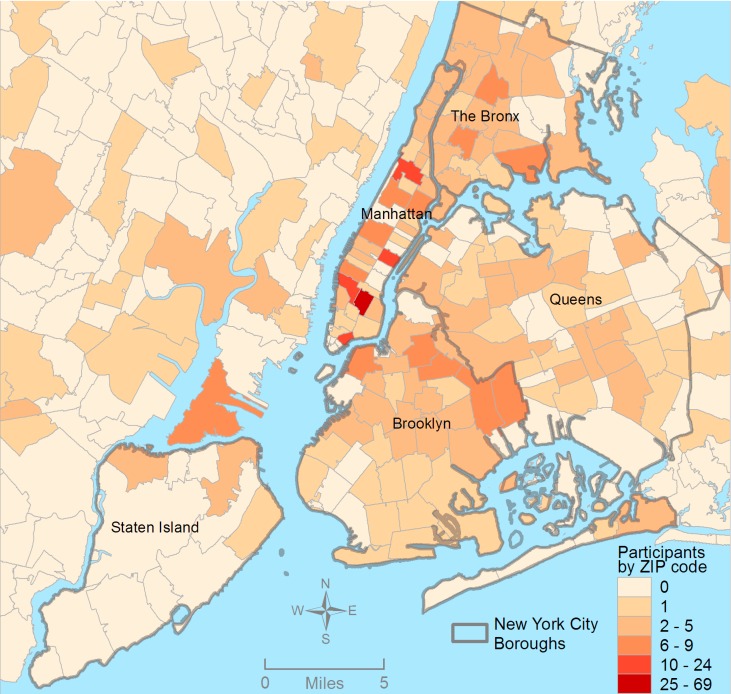
Project 18 participants by residential ZIP code, New York City metropolitan area.

First, we calculated the number of participants per residential ZIP code and used this information to create a map illustrating the distribution of residential neighborhoods for the Project 18 sample (See Figure 1). Second, descriptive analyses were conducted to present the distribution of neighborhoods (defined as boroughs) reported across all three contexts,* i.e.*, residential, social, and sex neighborhoods. Next, using these data, we examined concordance between borough types to assess the extent of spatial polygamy or differences between borough contexts. Specifically, we examined concordance between: (1) residential and social boroughs; (2) residential and sex boroughs; (3) social and sex boroughs; and (4) residential, social, and sex boroughs. Bivariable analyses were conducted to examine the influence of sociodemographic characteristics, psychosocial factors, social network characteristics, and health behaviors in relation to borough concordance, using Chi-Square statistics. Thus, all individual-level variables were examined separately in relation to borough concordance. Statistical significance was determined by *p* < 0.05. Statistical Package for the Social Sciences (SPSS) version 21 (SPSS IBM, New York, USA) was used to perform the statistical analysis.

## 3. Results

Figure 1 shows the spatial distribution of this sample of YMSM, by ZIP code, across the greater New York City metropolitan area. Participants resided in 122 of the 181 ZIP codes in New York City. The vast majority of the sample reported residing in the New York City metropolitan area with 69% of the sample reporting residence in one of the five New York City boroughs (Table 1). Of the five New York City boroughs, 32% and 15% of participants reported residence in Manhattan or Brooklyn, respectively, while approximately 10% reported either The Bronx or Queens as their borough of residence. Only 1% of the sample reported residing in Staten Island. With regard to socializing boroughs, 47% and 14% of this sample identified Manhattan or Brooklyn as their main boroughs for engaging in social activities. Overall, the distribution of sex boroughs more closely resembled the distribution of residential boroughs rather than socializing boroughs. In terms of borough concordance, approximately one third of YMSM in this sample reported discordant residential/social, residential/sex, and socializing/sex boroughs. In addition, 75% of participants in this study reported discordance across all three borough types (*i.e.*, residential, socializing, and sex boroughs). 

**Table 1:** Distribution of self-reported residential, social and sex neighborhoods (boroughs) among YMSM in the Project 18 study, 2009–2011.

Neighborhoods	% (N)
**Residential boroughs**	
Manhattan	32 (194)
Brooklyn	15 (91)
The Bronx	10 (59)
Queens	11 (66)
Staten Island	1 (4)
New York (Non-metro NYC area)	11 (67)
New Jersey	9 (53)
Connecticut	1 (7)
Other	9 (52)
**Socializing boroughs**	
Manhattan	47 (278)
Brooklyn	14 (83)
The Bronx	6 (37)
Queens	8 (47)
Staten Island	1 (3)
New York (Non-metro NYC area)	10 (61)
New Jersey	6 (37)
Connecticut	1 (4)
Other	7 (43)
**Sex boroughs**	
Manhattan	32 (191)
Brooklyn	14 (84)
The Bronx	10 (59)
Queens	9 (53)
Staten Island	1 (3)
New York (Non-metro NYC area)	10 (61)
New Jersey	8 (49)
Connecticut	1 (5)
Other	8 (49)
**Residential and social borough concordance**	
Yes	66 (395)
No	34 (203)
**Residential and sex borough concordance**	
Yes	68 (408)
No	32 (190)
**Social and sex borough concordance**	
Yes	63 (378)
No	37 (220)
**Residential, social, and sex borough concordance**	
Yes	25 (150)
No	75 (448)

Bivariable analyses indicated several notable differences in borough concordance by certain sociodemographic characteristics, psychosocial factors, social support network characteristics, as well as health behaviors (Table 2). First, Hispanic and Black YMSM were more likely to report borough discordance for all types of boroughs, whereas White YMSM were significantly more likely to report borough concordance, also across all borough concordance measures. For example, White YMSM were significantly more likely to report residential/socializing/sex borough concordance (46%) compared to Hispanic (29%) and Black (13%) YMSM (*p* < 0.001). Next, those reporting currently being enrolled in school reported more residential/social borough concordance (*p* < 0.05). Perceived familial socioeconomic status was also associated with borough concordance, with YMSM who reported a higher perceived socioeconomic status being more likely to report borough discordance while those reporting lower perceived socioeconomic status to be more likely to report borough concordance for socializing/sex and residential/socializing/sex boroughs. YMSM who reported residing with their parents were also more likely to report borough discordance across measures of residential/social, socializing/sex and residential/socializing/sex boroughs, while those who reported residing with their friends or a roommate reported more concordance for these borough types (all *p* < 0.05). Those who were exclusively homosexual reported more residential/social borough discordance (*p* < 0.01). With regard to psychosocial factors, YMSM who reported experiencing gay-related stigma in public forums were more likely to report discordant residential/social, socializing/sex and residential/socializing/sex boroughs (*p* < 0.05). YMSM who reported having come out to their peers were more likely to report concordant residential/sex and socializing/sex boroughs (both *p* < 0.05) while YMSM who reported greater gay community affinity were more likely to report discordant residential/social boroughs (*p* < 0.01). Regarding social support network characteristics, we found that greater frequency of communication with network members (≥weekly) was associated with less residential/social borough concordance (*p* < 0.05). In addition, YMSM who reported residential/socializing/sex borough concordance were more likely to report recent (last 30 days) alcohol use compared to those in discordant boroughs (88%* versus* 76%, *p* < 0.01). Self-reports of recent marijuana use were higher among those reporting both residential/socializing/sex as well as socializing/sex concordant boroughs (both *p* < 0.05). In terms of sexual behavior, YMSM in this study who reported concordance across all borough contexts were more likely to report engaging in condomless oral sex (all *p* < 0.05). 

## 4. Discussion

Spatial polygamy may be particularly salient in urban environments such as New York City due to the proximity of neighborhoods whose characteristics, norms, and cultures vary dramatically. In order to evaluate spatial polygamy among a sample of YMSM in New York City, we inquired about their home, social, and sex boroughs. In this study, we found that approximately two-thirds of participants reported concordance between residential/socializing, residential/sex, and sex/socializing boroughs, whereas 25% reported concordance between all three residential/socializing/sex boroughs. In addition, borough concordance varied by some sociodemographic characteristics, psychosocial factors, social support network characteristics, as well as certain health behaviors. 

**Table 2:** Bivariable associations between sociodemographic characteristics, psychosocial factors, social support network characteristics substance use and sexual behaviors and borough concordance for the Project 18 participants.

	Residential/Social Borough Concordance		Residential/Sex Borough Concordance		Social/Sex Borough Concordance		Residential/Social/Sex Borough Concordance	
	No	Yes	No	Yes	No	Yes	No	Yes
	(N = 203)	(N = 395)	(N = 190)	(N = 408)	(N = 220)	(N = 378)	(N = 448)	(N = 150)
	%	%	%	%	%	%	%	%
***Sociodemographic characteristics***								
**Race/ethnicity**								
Hispanic/Latino	44	36	41	37 ***	45	35 ***	42	29 ***
Black	20	17	23	15	21	15	19	13
Other (including Asian Pacific Islander)	11	14	17	12	16	12	14	12
White	25	33	20	36	18	38	25	46
**Currently enrolled in school** *(yes)*	81	88 *	84	87	85	86	84	89
**Perceived familial socioeconomic status**								
High	35	33	35	33	39	30 *	36	25 ***
Middle	39	36	37	37	37	37	39	33
Low	26	31	28	30	24	33	25	42
**Foreign-born** *(yes)*	10	11	12	11	10	12	12	9
**Household composition**								
Parents	61	50 *	61	51	64	48 **	65	22 ***
Friends/roommates	31	37	29	38	28	39	28	55
Other (e.g., shelter)	8	13	10	12	8	13	7	23
**Sexual identity** *(exclusively homosexual)*	50	37 **	42	41	44	40	42	41
**Currently in male-male relationship** *(yes)*	27	26	31	25	28	26	27	25
**Current housing status** *(unstably housed)*	6	5	9	4	5	5	6	3
***Psychosocial factors***								
**Ethnic identity** *(high)*	38	38	35	40	40	37	40	33
**Experienced public gay-related stigma** *(high)*	54	45 *	52	46	58	42 ***	55	28 ***
**Experienced personal gay-related stigma** *(high)*	47	49	44	50	47	48	49	45
**Has come out to peers** *(yes)*	71	67	59	74 ***	64	72 *	68	73
**Gay community affinity** *(high)*	51	38 **	41	43	45	41	42	43
**Internalized Homophobia** *(high)*	77	72	72	75	75	74	74	74
***Social support network characteristics***								
**Network size** *(>8 members)*	47	49	48	49	47	50	46	56
**Average duration of network relationships** *(≥2yrs)*	64	67	70	65	65	67	70	57
**Frequency of communication with network members** *(≥weekly)*	87	79 *	82	82	84	81	84	78
**Emotional support** *(high)*	82	83	73	72	73	72	73	68
**Material support** *(high)*	72	72	70	73	73	72	72	74
***Health behaviors***								
**Alcohol use**, last 30 days* (yes)*	79	80	78	80	77	81	76	88 **
**Marijuana use**. last 30 days* (yes)*	45	47	41	49	40	50 *	42	58 **
**Condomless oral sex**, last 30 days* (yes)*	48	58 *	48	58 *	43	61 ***	52	63 *
**Condomless anal sex**, last 30 days* (yes)*	19	21	20	21	20	21	21	17

Notes: * *p* < 0.05, ** *p* < 0.01, *** *p* < 0.001.

Our findings are similar to the existing research on spatial polygamy among adult MSM, including a recent study by Koblin and colleagues finding that few (15%) MSM in New York City had concordant home, social, and sexual neighborhoods; 31% men reported that none of their neighborhoods were the same. Similar to our study, previous research also showed that concordance of neighborhoods differed significantly by race/ethnicity. More specifically, a higher percentage of White men (22%) reported that all of the neighborhoods were the same compared to Black (10%) and Latino (11%) men, and a higher percentage of Black (35%) and Latino (37%) men reported that none of their neighborhoods were the same compared to their White counterparts (25%) [10]. However, the current study is the first study to examine spatial polygamy in YMSM and to evaluate the influence of multiple sociodemographic characteristics, psychosocial factors, social support network characteristics, and health behaviors in relation to neighborhood concordance. The Koblin study only examined and reported differences by race/ethnicity. Therefore, our study nicely builds on Koblin’s important work and provides a meaningful contribution to the literature. The present study examines adolescent and emerging adult MSM. 

There are several plausible explanations for our findings. Spatial polygamy may be common among our sample of New York City YMSM for a number of reasons, including the porosity of neighborhoods in New York City, cost of living differences by borough may mean that young people cannot necessarily live where they work, play, and go to school. In addition, there is easily accessible and multiple modes of public transportation between boroughs in New York City. Participants in our study might travel between home and work/school—which is supported by a recent study conducted among a sample of MSM in Baltimore [9]. As persistent residential segregation and its concomitant influence on amenities in neighborhoods have been documented [29,30,31,32], White YMSM may be more likely to report borough concordance because they may be more likely to live in neighborhoods with amenities that promote social activities—including parks and movie theaters; thus, they would not need to go to another borough to be socially-engaged. In addition, racial/ethnic minority (e.g., Black and Hispanic) men have a higher likelihood of not disclosing their sexual identity to their friends/family [17] and having more internalized homophobia [16], which may be a factor in less borough concordance (e.g., they might travel outside of their residential boroughs to socialize in neighborhoods where they can be more open about their sexuality). Moreover, people living with their parents might be less likely to be “out” and therefore report more discordant boroughs. It is possible that YMSM reporting lower perceived socioeconomic status are more likely to report borough concordance because they might not have the financial means to travel to different neighborhoods. Previous research has shown that urban sexual minority youth are exposed to LGBT hate crimes in their residential neighborhoods, which may be stress-inducing [33,34]; as such, YMSM experiencing gay-related stigma in public forums may be more likely to report discordant socializing/sex and residential/socializing/sex boroughs because they may be seeking to socialize in neighborhoods where they can be open about their sexuality and relationships. Our findings of some significant differences in mobility across boroughs (spatial polygamy) and social support network characteristics are in line with previous research among MSM populations [11]. In particular, we found that greater frequency of communication with network members was associated with less residential/social borough concordance and this may be because participants are socializing with social network members in neighborhoods outside of where they live. Interestingly, YMSM who reported residential/socializing/sex borough concordance were more likely to report recent (last 30 days) alcohol use, recent marijuana use, and recently engaging in condomless oral sex. There is literature on the health effects of gay enclaves, some of which suggests that among MSM, gay enclaves are associated with increased substance use [2,3] and increased high-risk sexual behavior [3,4]—perhaps due to social norms that encourage drug use and condomless sex.

## 5. Study Limitations

Several limitations of this study should be noted. First, as previously described, similar to other geographic locations, there are various ways to define neighborhoods in New York City—including based on one’s perception [10] as well as using ego-centric buffers [35,36,37,38,39], census block groups [40,41,42,43,44], census tracts [45,46,47,48,49], United Hospital Fund (UHF) defined neighborhoods [50,51,52,53,54], community districts [55,56,57,58,59], ZIP codes [60,61,62,63,64], and boroughs/counties [8,65,66,67,68]. In this study, we analyzed boroughs due to the structure of the data, which is a coarse neighborhood definition. For example, we were not able to evaluate neighborhoods within the borough/county of Manhattan—which consists of many geographic units previously used to define neighborhoods (e.g., in Manhattan there are 42 ZIP codes (using 2011 ZIP code boundaries), 287 census tracts (using 2010 census tract boundaries), and 1,092 census block groups (using 2010 census block group boundaries)—many of which have defined neighborhood names, including West Village, Chelsea, Kips Bay, Hell’s Kitchen, Midtown, Upper East Side, and Harlem). Because borough/county was used as the neighborhood definition, our study might underestimate spatial polygamy and we recognize there is potential for spatial misclassification [69], which may explain some of the null associations found in this study. However, in addition to New York City-based research [8,65,66,67,68], it should be noted that county as a neighborhood definition has been used in significant previous health-related research on neighborhoods in other geographic locations in general populations [70,71,72,73,74,75,76], as well as similar research in sexual minority populations [77,78,79,80,81,82]. Further, it should be noted that some studies have examined neighborhood-level exposures in New York City as the neighborhood definition and unit of analysis [83,84], which is an even coarser neighborhood scale than used in the current study. Second, because we restricted the neighborhood information to the first neighborhood name for each type (as there was often no data for the second and third neighborhood names for each neighborhood type and as it was not clear to us if the data were missing or not entered as participants may have only reported one or two neighborhoods), we recognize that spatial polygamy may be underestimated. Third, in this study, we did not examine neighborhoods where these young men went to school, sought medical care, worked and had other family, which may be salient to their lives. Fourth, the “uncertain geographic context problem” is a concern. The uncertain geographic context problem notes that a problem in neighborhood health research is spatial uncertainty in the actual areas that exert contextual influences on the individuals being studied and the temporal uncertainty in the timing and duration in which individuals experience contextual influences [85,86]. No measures of exposure by neighborhood (e.g., time spent in residential* versus* socializing neighborhood), for instance, were included in the study because this information was not included in the survey. In addition, there may be some under-reporting of substance use and condomless sex due to socially desirable responding. However, our use of audio computer-assisted self-interviews (ACASI) is a method of survey administration used to reduce this potential misclassification. Also, results were not corrected for multiple comparisons. Furthermore, our study was conducted among a sample of predominantly racial/ethnic minority HIV-negative YMSM in New York City. Consequently, the findings from this study might only be generalizable to racial/ethnic minority HIV-negative YMSM in urban settings. We have no reason to believe that the results would vary by HIV status, but it is possible that if one has had their HIV-positive status compromised in one neighborhood, they may avoid it altogether. Lastly, spatial sampling [87,88,89] was not utilized, so the recruitment strategy did not take into account the geographic distribution of New York City. However, we note that recruitment took place in all of the New York City boroughs/ other areas in the NYC metro area and the sample comes from the vast majority of the New York City metropolitan area.

## 6. Future Research and Study Implications

Future research should continue to examine spatial polygamy, including among YMSM populations, to understand salient spatial contexts including residential, social and sexual neighborhood contexts but also other contexts (not examined in the current study) such as school and work neighborhood contexts—and including the actual characteristics of the various neighborhoods such median household income, percent racial/ethnic minority, crime statistics and/or HIV rates. Future research can examine spatial polygamy using smaller neighborhood definitions—which might be more socially meaningful than the neighborhoods analyzed in the current study. As shown in previous research, qualitative [11] and web-based [10] methods can be used to examine spatial polygamy among MSM. Survey methods, as done in this study, can also be employed to examine spatial polygamy among MSM. However, more nuanced and specific information can come from geocoded data on frequently traveled spatial contexts using Global Positioning Systems (GPS) technology, which also have a temporal dimension [90,91]. Any potential GPS research can help minimize the concerns related to the uncertain geographic context problem [85,86]. Furthermore, future research studies could examine correlates and effect of spatial polygamy. For instance, one could test whether spatial polygamy could be the result of an individual moving to a new neighborhood—leaving friends and sex partners in their old neighborhood. More broadly, future research can examine additional sociodemographic characteristics, psychosocial factors, social network characteristics, and health behaviors and examine the geographic location of an individual’s social network members. Additional and more specific information on associations between sociodemographic characteristics, psychosocial factors, social network characteristics, and health behaviors associated with spatial polygamy may increase understanding of the most appropriate locations for targeted disease prevention and health promotion interventions (e.g., HIV prevention interventions). This is especially warranted given the persistence of stark disparities in HIV between non-sexual minority and sexual minority (e.g., MSM) populations, as well as socioeconomic and racial/ethnic disparities in HIV/AIDS among YMSM [92,93,94]. Findings from this and other studies on spatial polygamy in conjunction with emerging research showing HIV “hot spots” (*i.e.*, that HIV clusters spatially) [5,95,96,97,98] can be used to target HIV prevention strategies among MSM populations. Such targeted HIV prevention strategies should not only take into account potential HIV ‘hot spots’, but also consider other emerging work showing that sustained high viral load and durably suppressed viral load among HIV-infected individuals varies by neighborhood [68]. One study demonstrated that the percentage of HIV-infected New Yorkers varies by New York City borough and that certain boroughs (e.g., The Bronx) are more likely to have sustained high viral loads [68]. We highlight the importance of targeted HIV prevention and control programs because lower socioeconomic status MSM (who also in this study are more likely to be Black or Hispanic) have sex and live in neighborhoods, which are also likely to be low-income and as result probably have greater rates of untreated unsuppressed HIV. Thus, despite some evidence of Black YMSM not actually engaging in more condomless sexual acts [18,99], they have a higher likelihood of having sex with a man who is virally unsuppressed because he resides in a lower-income neighborhood. 

## 7. Conclusions

These findings suggest that spatial polygamy, or an individual moving across and experiencing multiple neighborhood contexts, is prevalent among urban YMSM and that spatial polygamy varies by multiple individual-level characteristics. Future research among YMSM populations should consider multiple neighborhood contexts in order to provide a more nuanced understanding of how and which neighborhood contexts influence the health and well-being of YMSM. This further examination of spatial polygamy (and individual-level characteristics associated with it) may increase understanding of the most appropriate locations for targeted disease prevention and health promotion interventions (e.g., HIV prevention interventions).
